# Independent and collective roles of surface structures at different length scales on pool boiling heat transfer

**DOI:** 10.1038/srep37044

**Published:** 2016-11-14

**Authors:** Calvin H. Li, Russell P. Rioux

**Affiliations:** 1 Department of Mechanical Engineering Villanova University, Villanova, PA 19085,USA.

## Abstract

Spherical Cu nanocavity surfaces are synthesized to examine the individual role of contact angles in connecting lateral Rayleigh-Taylor wavelength to vertical Kevin-Helmholtz wavelength on hydrodynamic instability for the onset of pool boiling Critical Heat Flux (CHF). Solid and porous Cu pillar surfaces are sintered to investigate the individual role of pillar structure pitch at millimeter scale, named as module wavelength, on hydrodynamic instability at CHF. Last, spherical Cu nanocavities are coated on the porous Cu pillars to create a multiscale Cu structure, which is studied to examine the collective role and relative significance of contact angles and module wavelength on hydrodynamic instability at CHF, and the results indicate that module wavelength plays the dominant role on hydrodynamic instability at CHF when the height of surface structures is equal or above ¼ Kelvin-Helmholtz wavelength. Pool boiling Heat Transfer Coefficient (HTC) enhancements on spherical Cu nanocavity surfaces, solid and porous Cu pillar surfaces, and the integrated multiscale structure have been investigated, too. The experimental results reveal that the nanostructures and porous pillar structures can be combined together to achieve even higher enhancement of HTC than that of individual structures.

Pool boiling study is of fundamental importance in understanding liquid-vapor phase change physics^1^,^2^,^3^,^4^,^5^ and enabling many important applications^6^,^7^. In pool boiling heat transfer study, two benchmarks are used to evaluate liquid-vapor phase change heat transfer performance: Critical Heat Flux (CHF) and Heat Transfer Coefficient (HTC)^8^. CHF is located at the peak of a boiling curve in the nucleate pool boiling regime, and the onset of CHF is accompanied by a vapor blanket formation that covers the whole heating surface and separates liquid from reaching the bottom heating surface^9^,^10^. Currently, the understanding of CHF mechanisms focuses on hydrodynamic instability model^11^,^12^,^13^ and revised models^14^,^15^,^16^,^17^ among others^18^,^19^,^20^,^21^,^22^. The hydrodynamic instability model investigated by Kutateladze^11^, Zuber^12^, and Lienhard and Dhir^13^ hypothesizes that when the velocity of vapor in escaping columns reaches a critical value, the interface wave on vapor columns will reach a Kevin-Helmholtz (K-H) wavelength, *λ*_*K*−*H*_, and cause a collapse of neighboring vapor columns. The weakness of this model is the failure to take into consideration of heating surface effects on hydrodynamic instability. Recent pool boiling studies on micro/nanoscale surface structures^23^,^24^,^25^,^26^,^27^,^28^,^29^,^30^ have led to the latest modifications of the hydrodynamic instability model with heating surface effects, including the contact angles^14^,^15^, surface wettability, and capillary wicking^16^,^17^. On the other hand, the study of hydrodynamic instability models on porous structures has been pursued, too, which focuses on controlling hydrodynamic instability wavelength by the characteristic length of porous structures of micron and millimeter scales^26^,^31^,^32^. It has been proposed that the hydrodynamic instability wavelength can be controlled by pore size, *d*, and surface pore distribution through porosity, *ε*, *as* 
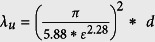
 on a flat porous structure^31^, and the pitch of porous cones as 
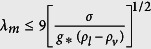
26,32, where *σ* is surface tension, *g* is gravitational acceleration, *ρ*_*l*_ and *ρ*_*v*_ are densities of liquid and vapor.

In this study, we will investigate the control mechanisms of hydrodynamic instability wavelength at different surface structure characteristic length scales, and will answer the following questions as (1) What are the individual and collective roles of contact angle with nanostructures, module wavelength of porous structures, and both together on controlling hydrodynamic instability wavelength for CHF enhancement? And (2) what are the individual and collective roles of nanostructures, porous structures, and both together on enhancing HTC ?

## Results and Discussion

### Spherical Cu nanocavity surfaces

**Figure 1:**
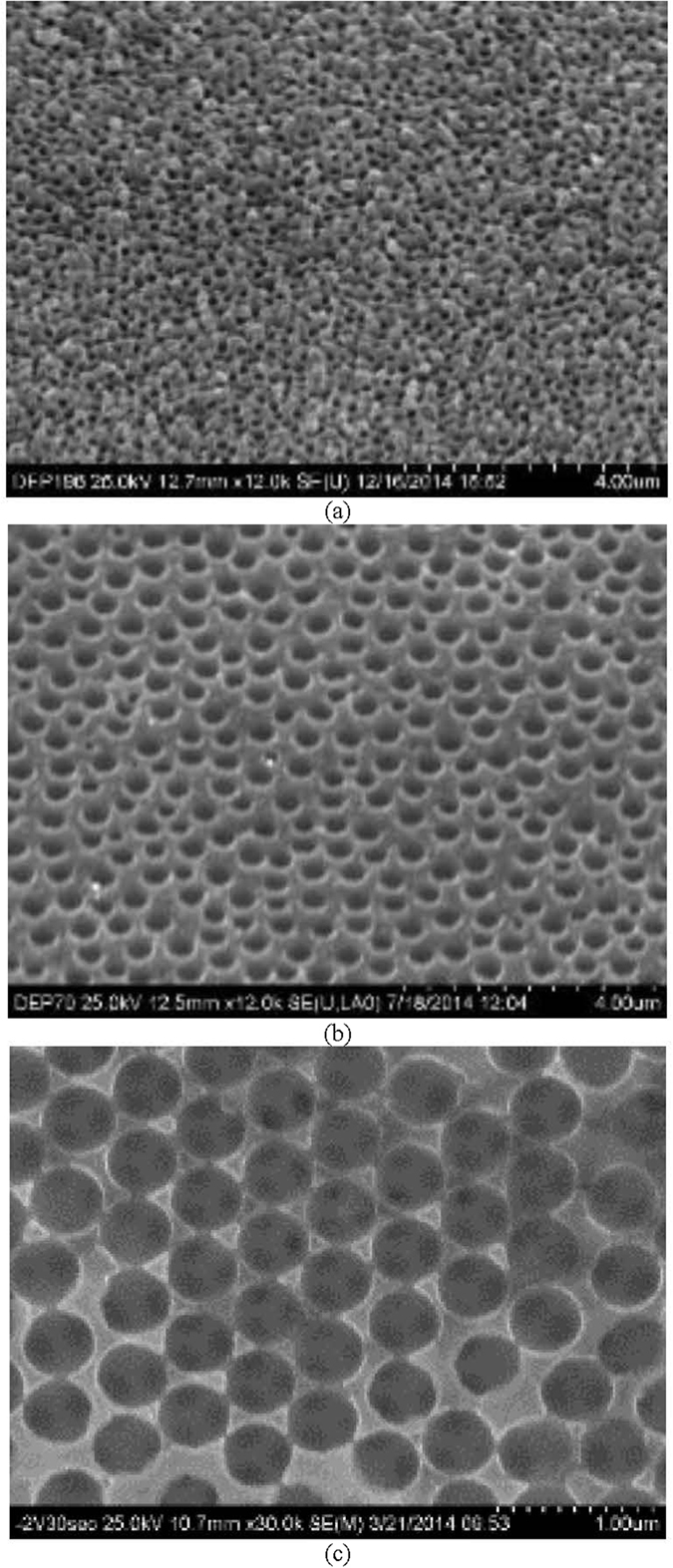
30° titled top view SEM images of (**a**) 200 nm and (**b**) 500 nm diameter spherical Cu nanocavity surfaces, and (**c**) top view of local pattern of spherical Cu nanocavities.

In order to investigate the individual role of contact angles on controlling hydrodynamic instability wavelength, uniform 200 nm and 500 nm diameter spherical Cu nanocavity surfaces have been synthesized (Fig. 1) to create different contact angles from that on a plain Cu surface, which enables to examine the magnitude of CHF enhancements due to the hydrodynamic instability wavelengths changed by contact angles. Equilibrium contact angles (measured when a droplet is not about to move), advancing contact angles (measured when a droplet is about to replace vapor/gas phase in front of it), and receding contact angles (measured when a droplet is about to give way to vapor/gas phase in front of it), are measured by a standard contact angle goniometer system^26^,^30^ on a plain Cu surface, 200 nm, and 500 nm diameter spherical Cu nanocavity surfaces. The smooth plain Cu surface has an average equilibrium contact angle of 78.35° with a standard deviation of 1.13°. Advancing and receding contact angles average at 85.65° and 40.01°, with standard deviations of 0.67° and 2.39°, respectively. For the 200 nm diameter spherical Cu nanocavity surface, the average equilibrium, advancing, and receding contact angles are measured to be 112.17°, 130.22°, and 36.4°, and their standard deviations are measured to be 4.84°, 2.25°, and 6.6°, respectively. And for the 500 nm diameter spherical Cu nanocavity surface, the average equilibrium, advancing, and receding contact angles are 122.41°, 146.44° and 35.48° with standard deviations of 3.45°, 2.38° and 4.42°, respectively. By structuring spherical Cu nanocavity surfaces of two different diameters, the contact angles have been significantly deviated from that on a plain Cu surface, and the impact on the hydrodynamic instability wavelength from the contact angles by nanostructured surfaces can be cross-checked along with other experimental reports in the literature. For example, between a plain Cu surface and the 500 nm spherical Cu nanocavity surface, equilibrium contact angles change from 78.35° to 122.41°, advancing contact angles change from 85.65° to 146.44°, and receding contact angles change from 40.01° to 35.48°. A detailed comparison among contact angles of these three different surfaces is presented in Table 1.

**Figure 2:**
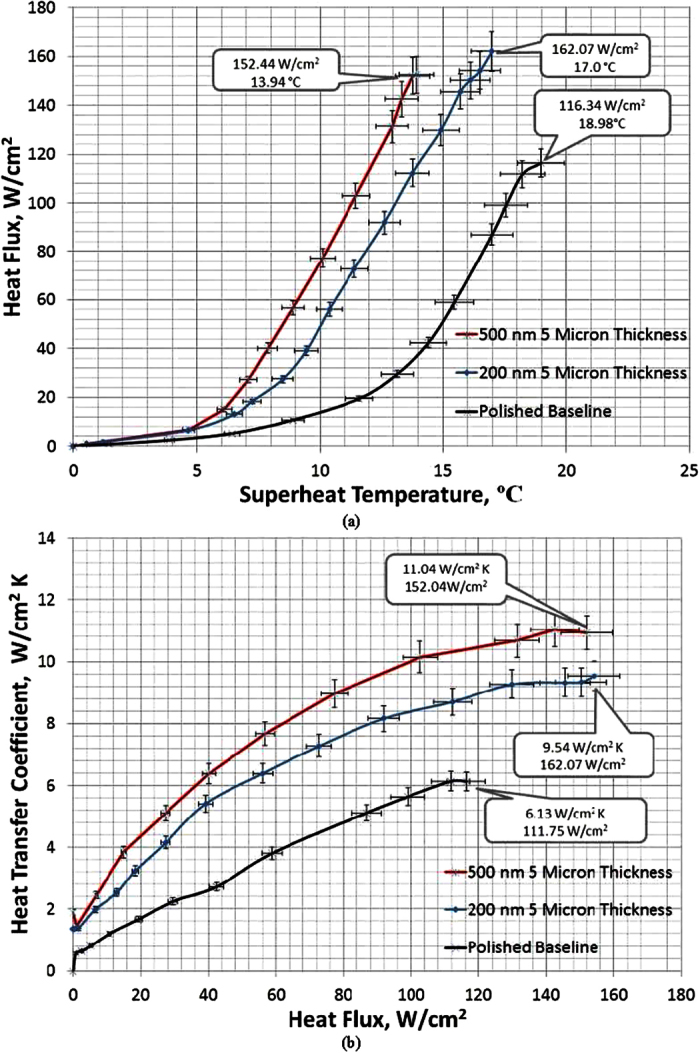
The boiling curves of a plain Cu surface, a 200 nm and a 500 nm diameter spherical Cu nanocavity structured surfaces. (**a**) heat flux vs. superheat temperature, and the inserts are CHF and its corresponding superheat temperature; and (**b**) heat transfer coefficient vs. heat flux, and the peak HTC and its corresponding heat flux.

In conducting the pool boiling heat transfer tests, the plain Cu surface is tested first to serve as a baseline for CHF and HTC, which have good agreements with the results of pool boiling on a plain Cu surface in literature^15^,^16^,^29^. As shown in Fig. 2, a CHF at 123.23 W/cm^2^ occurs on the plain Cu surface at a surface superheat temperature of 20.54 °C, at which the maximum HTC of ~6.00 W/(cm^2^K) also occurs. The CHF has been compared with some existing theoretical models^14^,^16^,^22^ and is listed in Table 2. The experimental study on spherical nanocavity surfaces is conducted to quantify CHF enhancements by contact angle reduction, and more importantly, to explore the mechanism of how contact angle determines CHF through the change of Kevin-Helmholtz wavelength (hydrodynamic instability wavelength). As shown in Fig. 2, with a layer of 5 μm thick spherical Cu nanocavity coating, the CHF has been increased to 152.44 W/cm^2^ and 162.07 W/cm^2^ on 500 nm and 200 nm diameter spherical Cu nanocavity surfaces, respectively, which are 36% and 45% higher than that of a plain Cu surface. The CHFs on 200 nm and 500 nm spherical Cu nanocavity surfaces are very close to each other, which could be caused by the almost identical receding contact angles of both surfaces. Meanwhile, there is a dramatic shift of boiling curves to the left on both spherical Cu nanocavity surfaces, as shown in Fig. 2(a), including the lower superheat temperatures at which CHFs happen. Interestingly, the boiling curve of 500 nm diameter spherical Cu nanocavity surface shifts further left to the boiling curve of 200 nm diameter spherical Cu nanocavity surface, which means a higher heat flux at a lower superheat temperature on 500 nm diameter spherical Cu nanocavity surface, or a higher HTC at a given superheat temperature/heat flux. This can be better represented by the HTC curves in Fig. 2(b).

**Figure 3:**
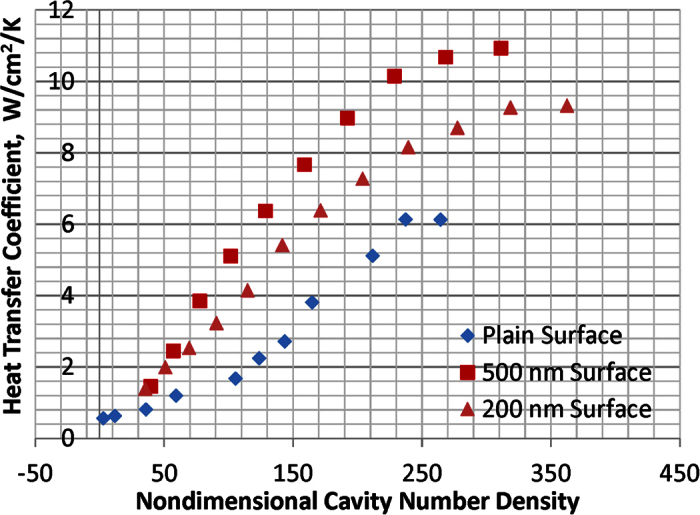
HTC and Na vs. superheat temperature.

The HTCs have been significantly increased over the whole boiling process for both spherical Cu nanocavity surfaces compared to that of a plain Cu surface, and peak at 11.04 W/cm^2^K on 500 nm diameter nanocavity surface and at 9.54 W/cm^2^K on 200 nm diameter nanocavity surface. The HTCs are determined by contact angle and cavity size on nanocavity surfaces. According to the heterogeneous nucleation theory^33^, a bigger contact angle will promote bubble nucleation at a lower superheat temperature. With equilibrium contact angles at 78.35^o^ on a plain Cu surface, 122.41^o^ on 500 nm spherical Cu nanocavity surface, and 112.17^o^ on 200 nm spherical Cu nanocavity surface, the required bubble nucleation superheat temperature will be lower and the active nucleation density will be higher at a bigger contact angle. Hence, with bigger equilibrium contact angles on both spherical Cu nanocavity surfaces, the pool boiling curves on those two surfaces have shifted to the left by a higher active nucleation site density at a lowered superheat temperature for bubble nucleation^33^. As a result, HTCs have been increased on both 200 nm and 500 nm spherical Cu nanocavity surfaces, with the highest HTC on 500 nm spherical Cu nanocavity surface. This relationship has been well represented in the model by Basu, Warrier and Dhir^34^ that active nucleation site density, Na, is a function of contact angle and wall superheat temperature, as shown in Equation 1. The dimensionless active nucleation site densities of a plain Cu surface, the 200 nm and 500 nm diameter spherical Cu nanocavity surfaces have been calculated and plotted with the experimental HTCs of the three surfaces in Fig. 3. It is clearly illustrated in Equation 2 33 that contact angle affects HTC through an active nucleation site density, *Na*, and the slopes of HTC curves are a function of bubble departure diameter and frequency.


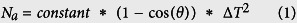



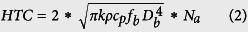


where *θ* is contact angle, Δ*T* is superheat temperature, *k* is liquid thermal conductivity, *ρ* is vapor density, *c*_*p*_ is vapor specific heat, *f*_*b*_ is bubble departure frequency, and *D*_*b*_ is bubble departure diameter.

To understand the enhanced CHFs with reduced contact angles on spherical Cu nanocavity surfaces, the theoretical models by Kandlikar^14^ and Chu *et al*.^16^ have been used to compare the experimental and theoretical CHFs first, which predict CHFs at 133.11 W/cm^2^ and 89.75 W/cm^2^ based on the measured receding contact angle of 40° on a plain Cu surface in this study, respectively. These two theoretical models replace the constant in Zuber’s hydrodynamic instability model through a correction factor function of receding contact angle based on a force balance along the heating surface, rather than changing the hydrodynamic instability wavelength. And a contrast between experimental and predicted CHFs of spherical Cu nanocavity surfaces shows some significant difference in this study. The difference might be caused by many reasons, but one of the possible reasons as seen by this study is the lack of direct connection between contact angle and hydrodynamic instability wavelength. In Zuber’s model, the critical Kevin-Helmholtz wavelength of hydrodynamic instability in the vertical direction of gravity is a function of the Rayleigh-Tayler wavelength, which decides the spacing and diameter of an array of vapor columns in lateral direction on a heating surface. Just modifying the constant with a factor function of contact angle in Zuber’s model could not really answer the question of how the contact angle changes the Rayleigh-Tayler wavelength or directly the Kevin-Helmholtz wavelength on hydrodynamic instability. By revisiting the theoretical development of Zuber’s model^12^ on a plain surface, it states that the velocity of escaping vapor is a function of Kevin-Helmholtz wavelength, *λ*_*K*−*H*_ in Equation 3, and surface area ratio taken by vapor columns on a heating surface is a constant of *π*/16 in Equation 4.


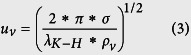



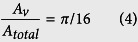


where *ρ*_*v*_ is vapor density, *h*_*lv*_ is latent heat, *u*_*v*_ is vapor velocity, *σ* is surface tension, *A*_*v*_ is the surface area taken by vapor column, and *A*_*total*_ is the total heating surface area. Here the Kevin-Helmholtz wavelength is determined by Rayleigh-Taylor wavelength as Equations 5 & 6,


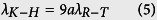


and the critical Rayleigh-Taylor wavelength is:


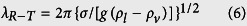


Where *a* = 1/4, *g* is gravitational acceleration, *ρ*_*l*_ and *ρ*_*v*_ are liquid and vapor densities.

**Figure 4:**
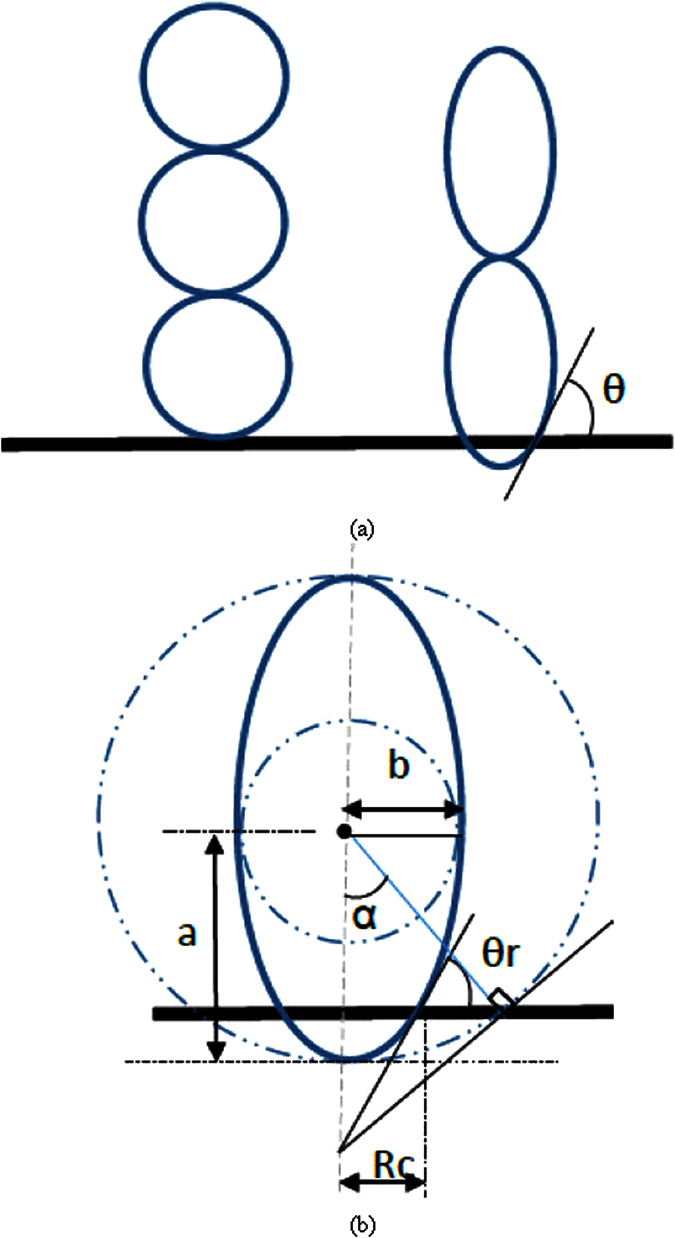
Sketch of the bubble shapes in vapor columns with different receding contact angle. (**a**) zero receding contact angle and non-zero receding contact angle bubble shapes; and (**b**) the geometric dimensions of an ellipse bubble with a receding contact angle of θr.

In predicting CHFs by hydrodynamic instability wavelength on a plain surface, the Kevin-Helmholtz wavelength (hydrodynamic instability wavelength) is exclusively coupled with the Rayleigh-Taylor wavelength in Zuber’s model. Hence, in this study, we hypothesize that the Kevin-Helmholtz wavelength on nanostructured surfaces is controlled by both the receding contact angle and the Rayleigh-Taylor wavelength. If the receding contact angle is zero, the vapor column will be formed by a series of spherical vapor bubbles one after another as shown in Fig. 4(a), and the Kevin-Helmholtz wavelength will be four times of vapor bubble radius, *r*, which is a quarter of the Rayleigh-Taylor wavelength, *λ*_*R*−*T*_ = 4 ∗ *r*. However, if the receding contact is not zero, a vapor bubble sitting on an active nucleation cavity will elongate into an ellipse shape, as shown in Fig. 4(b). The radius of nucleation cavity, *Rc*, will be a function of the short axis radius of the ellipse bubble, *b*, as *R*_*c*_ = *b* ∗ sin(*α*), and the distance between the heating surface to the center of the ellipse bubble will be a function of the long axis radius, *a*, as *b* = *a* ∗ cos(*α*). The geometric relationship among a*, b, Rc*, *α* and *θ*_*r*_ is *a* ∗ sin(*α*) ∗ tan(*α*) = *R*_*c*_ ∗ tan(*θ*_*r*_), or a*R_*c*_/cos(*α*) = *b*^2^ ∗ tan(*θ*_*r*_), where *θ*_*r*_ is receding contact angle and 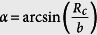. The Rayleigh-Taylor wavelength still determines the spacing among vapor columns, but is decoupled from the Kevin-Helmholtz wavelength.

Assuming the Kevin-Helmholtz wavelength is still four times of vapor ellipse long axis radius, *a*, (*λ*_*KH*_ = 4 ∗ *a*), while the short axis radius, *b*, is a quarter of the Rayleigh-Taylor wavelength, *λ*_*RT*,*c*_ = 4 ∗ *b*. The geometric relation between long axis, *a*, and short axis, *b*, in the elongated ellipse shape vapor bubble is *a* = *b*/(*constant* ∗ cot(*θ*_*r*_)), as shown in Fig. 2. The CHF on a nanostructured surface will be a function of receding contact angle as shown by Equation 7 based on Equations 3, 4 & 5, where *C* = *π*/32.


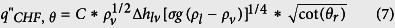


**Figure 5:**
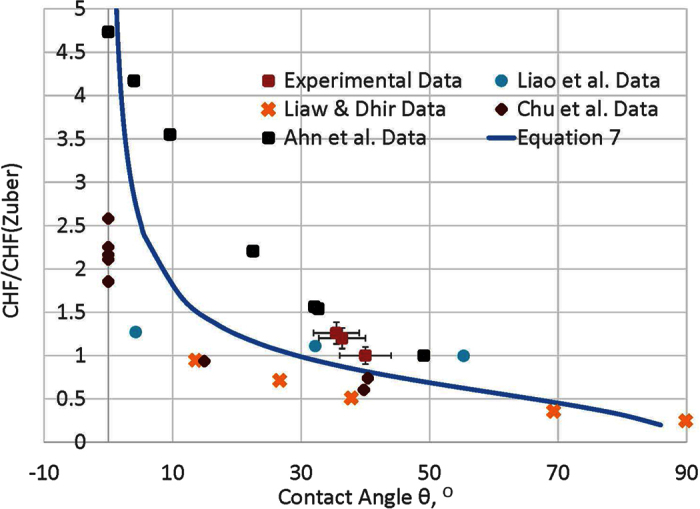
The relative CHFs of experimental data and predictions from Equation 8 over CHF predicted by Zuber vs. receding contact angle.

The relative experimental CHF enhancements of this study and four other different research groups^15^,^16^,^22^,^29^, and the predictions from Equation 7 based on the receding contact angles in the five experimental studies are presented in Fig. 5, which shows a comparable match between experimental data and the predictions of Equation 7. The physics behind the match illustrates the role receding contact angle has on coupling the Rayleigh-Taylor wavelength and the Kevin-Helmholtz wavelength in hydrodynamic instability models, in which the Kevin-Helmholtz wavelength decides the hydrodynamic instability of vapor columns, and is a function of both receding contact angle and the Rayleigh-Taylor wavelength.

### Solid and porous Cu pillar surfaces

**Figure 6:**
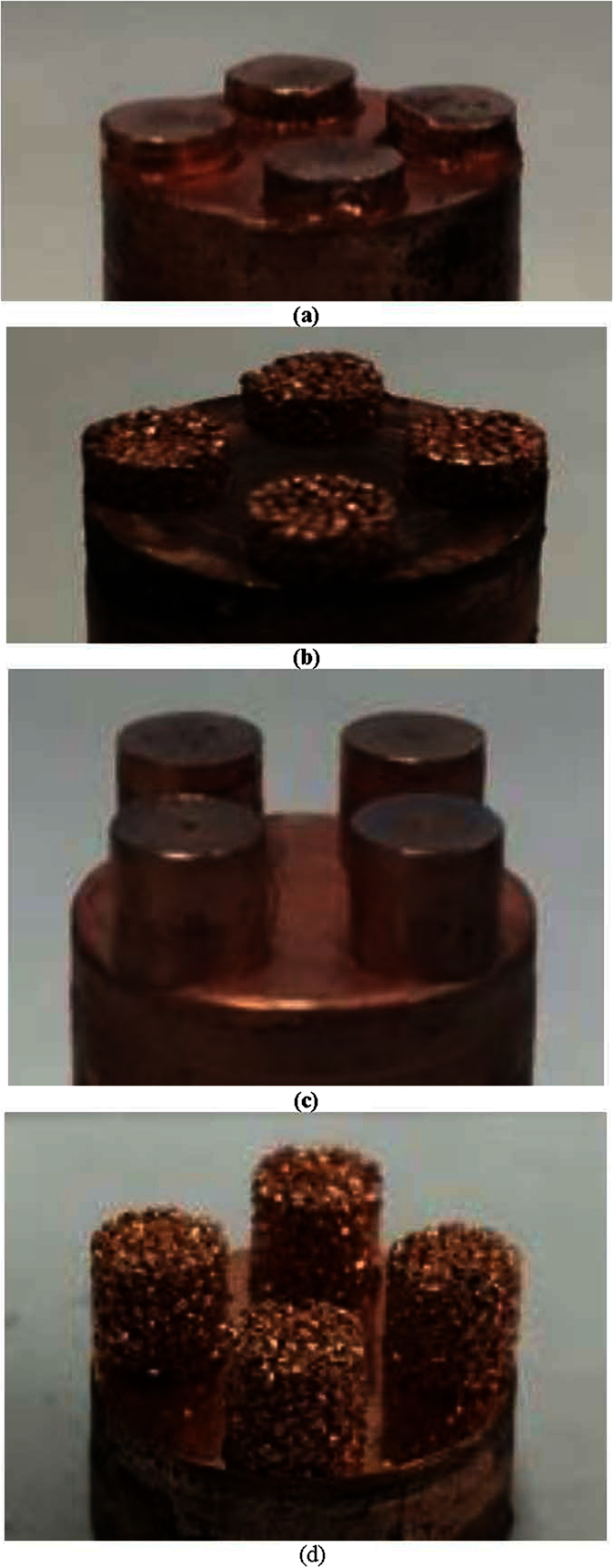
Photo images of solid and porous Cu pillar structured surfaces of ¼ length (**a & b**) and full length (**c & d**).

In order to further examine the surface structure effects on hydrodynamic instability at CHF, solid and porous Cu pillar surfaces have been created by the same sintering method reported in literature^26^,^35^, as shown in Fig. 6. More specifically, two effects are of interest: (1) The vertical distance from a heating surface where vapor columns collapse to form a vapor blanket at the onset of CHF, and (2) the relation between the Kevin-Helmholtz wavelength and porous surface structure characteristic size, module wavelength (the surface pore distribution or the pitch of modulated pillars), as demonstrated by the CHF study on flat porous structures^31^ and modulated porous structures^32^,^35^ that the Kevin-Helmholtz wavelength is a function of the characteristic size of a surface structure. According to hydrodynamic instability theory, neighboring vapor columns collapse to form a vapor blanket covering the whole plain heating surface at the onset of CHF. The study by Haramura and Katto^20^ proposes that approaching to the onset of CHF, a large vapor mushroom is formed on a heating surface by an array of small vapor jets existing in a liquid layer underneath the vapor mushroom. If the liquid layer underneath the vapor mushroom diminishes before the vapor mushroom departs, a vapor blanket on the whole heating surface will happen and the CHF occurs. This liquid layer is estimated at a height around ¼ Kevin-Helmholtz wavelength above the plain heating surface, which is also proposed in an earlier study by Gaertner^18^. Another study of the critical vapor film thickness at the onset of CHF by Tanaka^21^ proposes that the thickness for the vapor film is on the order of 0.1–1 mm. Hence, in this study both solid pillars shorter and taller than ¼ Kevin-Helmholtz wavelength are constructed on the heating surfaces to examine the effects of the required pillar length, so that it can effectively prevent the vapor blanket formation from the neighboring vapor columns collapsing. Furthermore, porous pillars at both lengths have been constructed, which could offer the liquid water downward flow channels inside the pillars to reach the bottom heating surface and sustain the liquid-vapor phase change on the bottom heating surface, in addition to physically preventing neighboring vapor columns from collapsing together as solid pillars do.

**Figure 7:**
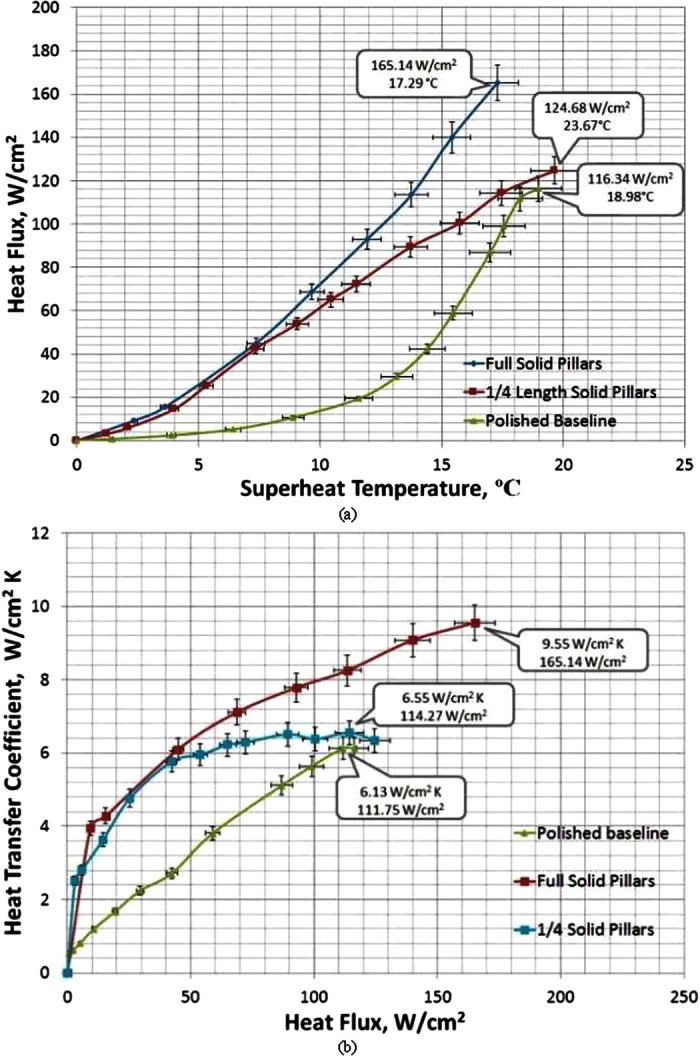
Boiling curves for ¼ length and full length solid Cu pillar surfaces. (**a**) heat flux vs. superheat temperature, and the inserts are CHF and its corresponding superheat temperature; and (**b**) heat transfer coefficient vs. heat flux, and the peak HTC and its corresponding heat flux.

As reported that the vapor blanket has a thickness of 0.1–1 mm at the onset of CHF^21^, the liquid layer beneath a hovering bubble has a thickness of ½ vapor bubble diameter^20^, water capillary length is 2.7 mm^28^, and water vapor Rayleigh-Taylor wavelength is 15.4 mm^13^, pool boiling heat transfer tests have been conducted on surfaces with both solid and porous Cu pillars of 4 mm diameter at two different lengths, 3.6 mm and 0.9 mm, on a 12.7 mm diameter Cu substrate to study pillar length effect and hydrodynamic instability wavelength determined by the module wavelength of 8.7 mm on Cu pillar surfaces. As shown in Fig. 7, the 3.6 mm length Cu pillars will be referred to as “Full Length” while the 0.9 mm length Cu pillars will be referred to as “1/4 Length”. The Cu powder used to synthesize the porous pillars has a size range of 550–600 μm, and the resulted pore sizes in the porous pillars are found to be in the range of 50 to 220 μm. The details of the Cu pillars synthesis process could be found in previous work^26^,^35^. The full length Cu pillars should be able to penetrate through the vapor blanket covering the bottom heating surface once CHF has been reached, but the ¼ length Cu pillars should be just comparable to the thickness of the vapor blanket, which can not effectively prevent neighboring vapor columns from collapsing together at the onset of CHF.

As illustrated by Fig. 7(a), for the ¼ length solid Cu pillar surface, CHF enhancement is negligible over that of a plain Cu surface due to the fact that the pillars are not tall enough to prevent the neighboring vapor columns from collapsing into a vapor blanket. Once the solid Cu pillars are tall enough, as in the case of the full length solid Cu pillar surface, they can interfere in the vapor column collapse and prevent the formation of a vapor blanket to block the liquid from reaching the bottom heating surface. The full length solid Cu pillars replace the Kevin-Helmholtz wavelength by the module wavelength to be the hydrodynamic instability wavelength on CHF, which means the full length solid Cu pillars will break the vapor blanket and allow the liquid to flow along the pillars’ side surface down to the bottom heating surface. As a result, the CHF of full length solid Cu pillar surface has been increased to 165.14 W/cm^2^ by 48% over that on a plain Cu surface. The CHF on ¼ length solid Cu pillar surface is virtually the same as that on a plain flat surface due to the fact that the ¼ length solid Cu pillars are too short to replace the Kevin-Helmholtz wavelength by the module wavelength for the hydrodynamic instability wavelength which decides the onset of CHF.

Meanwhile, the magnitude of the CHF enhancement on full length solid Cu pillar surface is the same as that on 500 nm diameter spherical Cu nanocavity surface, which means module wavelength of solid pillars has the same magnitude of influence on hydrodynamic instability as contact angles have. This comparison reveals that both surface structures, solid Cu pillars and Cu nanocavities, have the same individual magnitude influence on hydrodynamic instability wavelength at CHF.

**Figure 8:**
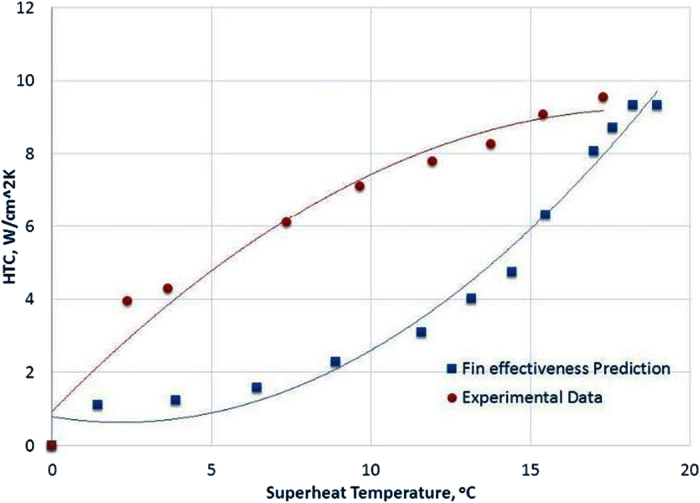
Comparison of HTCs of fin effectiveness prediction and experimental measurement on a full length solid Cu pillar surface.

As to the experimental HTCs, shown in Fig. 7(b), the highest HTC on the full length solid Cu pillar surface is 9.55 W/cm^2^K at the CHF, which is 56% higher than the highest HTC on a plain Cu surface. The highest HTC on ¼ length solid Cu pillar surface is identical to that of a plain Cu surface. The fact that ¼ length solid Cu pillar surface has the same CHF and HTC as that on a plain Cu surface confirms the conclusion once again that the same physical mechanism responds to the onset of CHF on both ¼ length solid Cu pillar surface and a plain Cu surface, resulting in the same CHF and peak HTC. The fin effectiveness of both ¼ and full length solid Cu pillar surfaces are examined as well in this study to understand the pool boiling heat transfer enhancements on both surfaces. The heat flux enhancement due to the extended surface area effect for ¼ length solid Cu pillar surface is calculated as negative in which the predicted fin effectiveness is 0.85. This predicted reduction is due to the extra thermal resistance of ¼ length solid Cu pillars and high HTC of boiling heat transfer^8^. While the fin effectiveness of full length solid Cu pillars can predict the experimental HTC value at the onset of CHF, the predicted trend of HTC on the full length solid Cu pillar surface by the fin effectiveness does not match the trend of experimental HTC on the surface, as shown in Fig. 8. The experimental HTC and heat flux at a given superheat temperature before the onset of CHF are higher than that predicted by fin effectiveness on the full length solid Cu pillar surface, which means the solid Cu pillars play an extra role of promoting a better bubble ebullition beyond just offering an extended surface area for the heat transfer enhancement.

**Figure 9:**
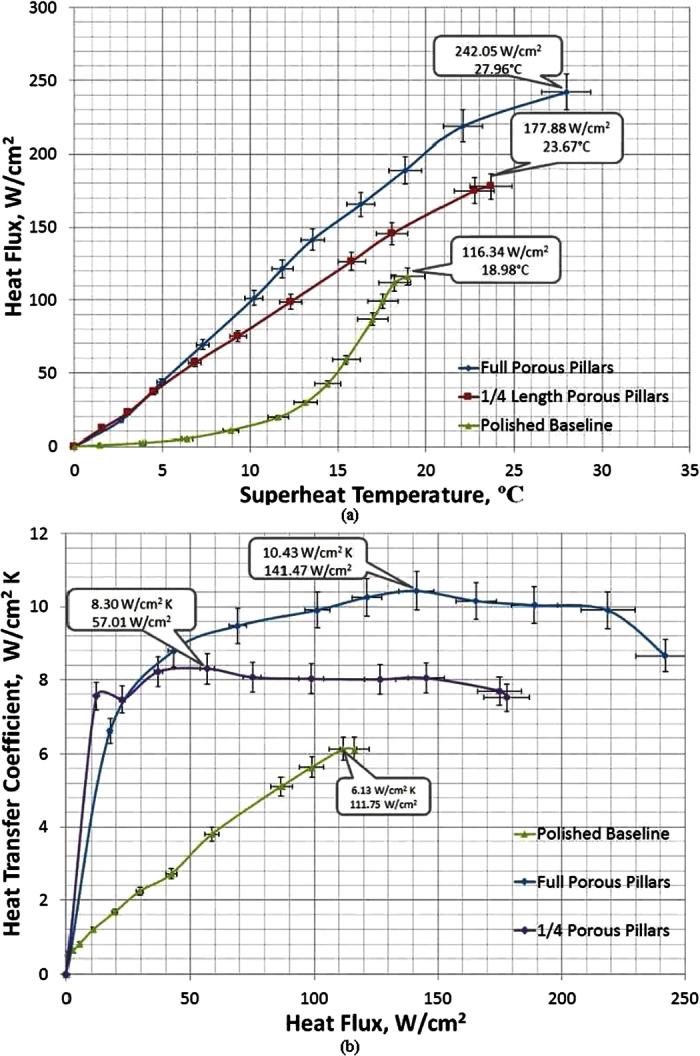
Boiling curves for ¼ length and full length porous Cu pillar surfaces. (**a**) heat flux vs. superheat temperature, and the inserts are CHF and its corresponding superheat temperature; and (**b**) heat transfer coefficient vs. heat flux, and the peak HTC and its corresponding heat flux.

In contrtast to the boiling performance on solid Cu pillar surfaces, as shown in Fig. 9(a), the CHFs of both ¼ length and full length porous Cu pillars surfaces have been significantly increased to 177.88 W/cm^2^ and 242.05 W/cm^2^, or 53% and 100% enhancements over that of a plain Cu surface, respectively. The experimental CHF enhancement by full length porous Cu pillar surface matches well with the prediction by Equation 3, as *CHF* ~ *u*, that the CHF of full length porous Cu pillar surface is 2 times of CHF on a plain Cu surface because the Kevin-Helmholtz wavelength of a plain Cu surface is *λ*_*K*−*H*_ = 34.65 mm, while the module wavelength of full length porous Cu pillar surface is *λ*_*module*_ = 8.7 mm and around 1/4 of *λ*_*K*−*H*_.

Based on previous experimental studies of pool boiling heat transfer of porous Cu pillar surfaces^26^,^35^ and the solid Cu pillar surface discussed above, the CHFs and HTCs of both ¼ length and full length porous Cu pillar surfaces have been examined to understand the liquid replenishment effect on CHF enhancement from the enabled downward liquid flow through the pores inside porous Cu pillars. For the ¼ length porous Cu pillar surface, the pillars can not effectively prevent neighboring vapor columns from collapsing together due to the fact that the height is comparable to the thickness of the vapor blanket. But the porous structure can enable the downward flow of liquid water inside of the pillars to reach the bottom heating surface when the liquid contacts the top of ¼ length porous Cu pillars due to the nature of the fluctuation in the vapor blanket thickness, which sustains the phase change heat transfer process to reach a higher CHF value.

Meanwhile, the HTCs of both porous Cu pillar surfaces quickly increase to the maximum HTC plateau at a low superheat temperature (Fig. 9(b)). This shift to a much lower superheat temperature at peak HTC is a distinctive difference between boiling curves on solid Cu pillar surfaces and porous Cu pillar surfaces. The HTCs on porous Cu pillar surfaces quickly increase to a maximum value and stay at that value over a plateau until CHFs have been reached. While the HTCs on solid Cu pillar surfaces gradually increase over the whole boiling process and reach peak values at the onset of CHF. The difference is caused by the fact that solid Cu pillars serve as an extended surface for heat transfer in the liquid water body only, while porous Cu pillars can promote bubble nucleation at an early stage of pool boiling. The sharp increase of HTC to its peak demonstrates that a significant amount of nucleation sites have been activated at a low superheat temperature. And the liquid water flowing inside the porous Cu pillars sustains the liquid mass flux demand of phase change process on those active nucleation sites until CHF has been reached.

**Figure 10:**
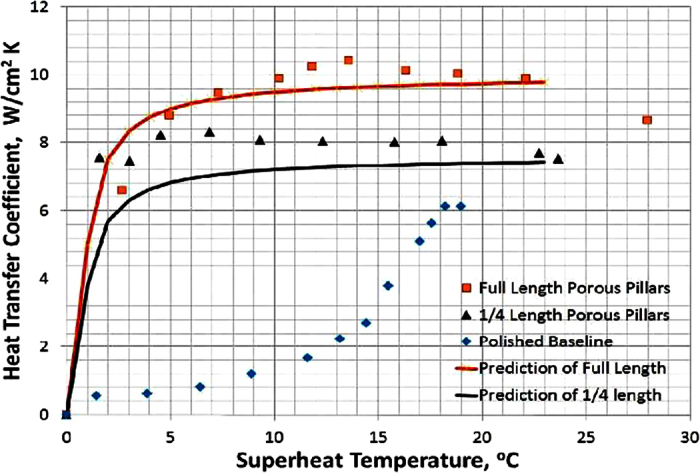
The experimental and predicted HTCs of porous Cu pillar surfaces vs. superheat temperature.

The experimental and theoretical studies of pool boiling heat transfer on a porous surface by Webb^35^ point out the heat transfer coefficient is a function of pore size and superheat temperature. Based on the experimental data from Liter, Li and others^20^,^32^, the HTC model of Webb can be modified as a function of superheat temperature, pore diameter, and porous structure thickness as shown in Equation 8.


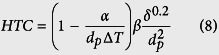


where *α* = *c* ∗ *σ* ∗ *T*_*sat*_ ∗ *v*_*v*_/*h*_*lv*_, 
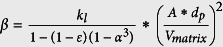
 is the geometry factor of the porosity *ε* as defined in appendix B of^19^, *k*_*l*_ is liquid thermal conductivity, *α* = *d*_*b*_/*d*_*p*_, *d*_*b*_ is bubble diameter, *d*_*p*_ is pore diameter and a function of particle diameter at micron scale, A is total surface area, *V*_*matrix*_ is the volume of porous structure, and *δ* is the thickness of porous structure. If the thickness, *δ*, can be approximated by half of the porous pillar length, then the HTCs of both full length porous Cu pillar surface and ¼ length porous Cu pillar surface can be predicted and compared in Fig. 10.

The peak HTCs at low surface superheat temperatures on both ¼ length and full length porous Cu pillar surfaces further illustrate the importance of the increased active nucleation site density that is offered by the porous Cu pillars, which is similar to the reason for higher HTCs on spherical Cu nanocavity surfaces compared to that on a plain Cu surface. The totally different active nucleation site size scales of the two different types of surfaces, one is of nanometer size and the other is of micron size, present two different trends of HTCs on the nanocavity surfaces and porous pillar surfaces.

### Integrated structure of 500 nm spherical Cu nanocavity coated full length porous Cu pillars

**Figure 11:**
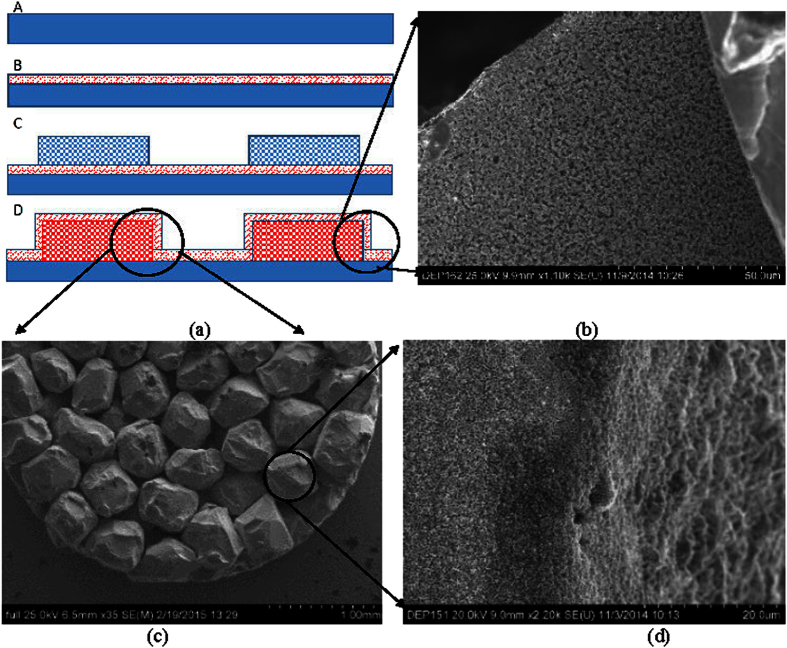
Integrated surface wettability and hydrodynamic instability control. (**a**) Procedure of integration: step A, smooth plain Cu surface polishing; step B, 500 nm diameter spherical Cu nanocavicity coating; step C, porous Cu pillar synthesizing; and step D, Spherical Cu nanocavity layer on the surfaces of porous Cu pillar integration; (**b**) SEM image of bottom Cu nanocavity coating; (**c**) SEM image of porous Cu pillars; and (**d**) the Cu nanocavity coating on the microparitcle surface of porous Cu pillars.

Based on the experimental results of the individual pool boiling tests on Cu nanocavity surfaces and Cu pillar surfaces, two questions to be answered in the next study are: (1) Can the CHF be further enhanced by integrating nanocavities into porous pillar structures? And (2) can the HTC be further enhanced by integrating nanocavities into porous pillar structures? Therefore, in order to understand the collective roles on CHT and HTC from surface structures of both scales, an integrated surface of 500 nm diameter spherical Cu nanocavity coated full length porous Cu pillars has been created to examine the potential for even greater CHF enhancement, as shown in Fig. 11.

**Figure 12:**
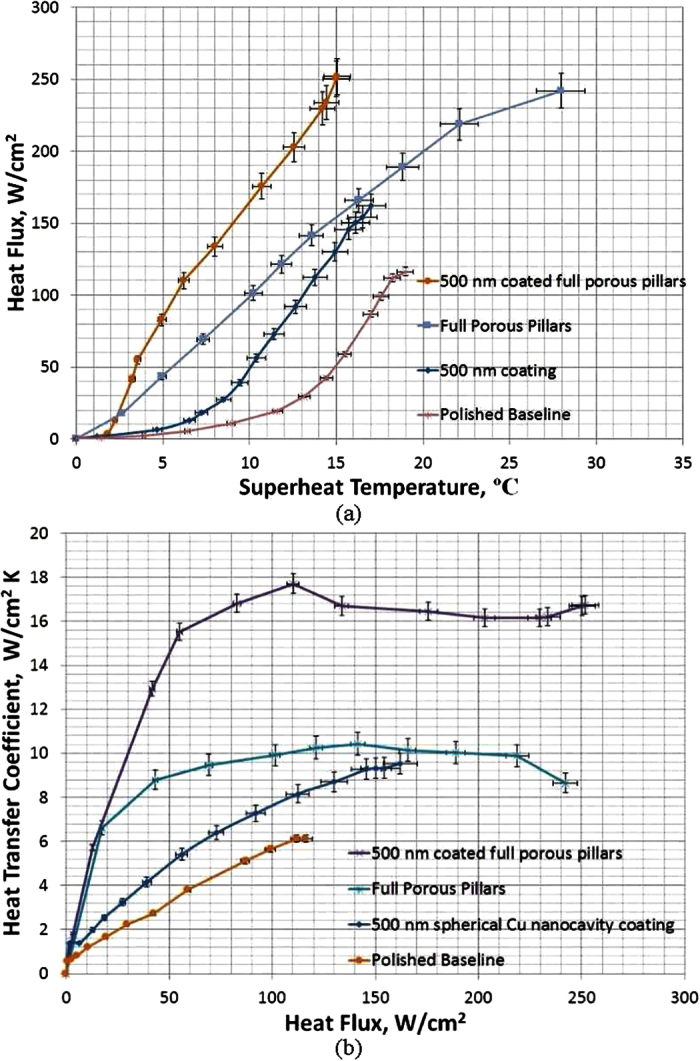
Boiling curves for a plain Cu surface, 500 nm diameter spherical Cu nanocavity surface, full length porous Cu pillar surface, and integrated 500 nm spherical Cu nanocavity with full length porous Cu pillar surfaces. (**a**) heat flux vs. superheat temperature, and the inserts are CHF and its corresponding superheat temperature; and (**b**) heat transfer coefficient vs. heat flux, and the peak HTC and its corresponding heat flux.

However, it is quite interesting to see in the test that the CHF of the integrated structure of 500 nm spherical Cu nanocavity coated full length porous Cu pillars (named as 500 nm coated full porous pillar surface in Fig. 12(a)) is 251.73 W/cm^2^, which is virtually the same as the CHF of 242.05 W/cm^2^ on the uncoated full length porous Cu pillar surface (named as full porous pillars in Fig. 12(a)). The only noticeable contribution from the 500 nm spherical Cu nanocavity coating is the dramatically reduced superheat temperature at which the CHF happens, 15.04 ^o^C from the 27.96 ^o^C on the uncoated full length porous Cu pillar surface.

Even though it comes as a surprise that CHF has not been further enhanced, it could be well explained by the physics that both nanocavity and porous pillar structures are trying to modify the hydrodynamic instability wavelength. The contact angle on the nanocavity surface tries to modify the hydrodynamic instability wavelength based on Rayleigh-Taylor wavelength, while the porous pillar surface replaces the hydrodynamic instability wavelength by the module wavelength. As argued by Polezhaev, Yu V., and S. A. Kovalev^18^, Liter and Kaviany^19^, and the analysis in the section of porous pillar surface test of this study, the module wavelength of pillar surfaces will dominate the CHF enhancement over contact angle on hydrodynamic instability wavelength when both length scales are presented.

While the CHF enhancement is dominated by porous Cu pillars, the HTC has been significantly enhanced by combining 500 nm spherical Cu nanocavity coating with porous Cu pillars. As shown in Fig. 12(b), the HTC curve of the 500 nm spherical Cu nanocavity coated full length porous Cu pillar surface has a sharp increase, the same as observed on porous Cu pillar surfaces, but to a much higher plateau value. The peak HTC of the spherical Cu nanocavity coated full length porous Cu pillar surface is 17.7 W/cm^2^K, compared to 10.43 W/cm^2^K on the uncoated full length porous Cu pillar surface and 8 W/cm^2^K on the 500 nm spherical Cu nanocavity surface at the same heat flux of 110 W/cm^2^. This peak HTC on the combined surface is identical to the sum of the individual HTCs from the 500 nm spherical Cu nanocavity surface and the full length porous Cu pillar surface, which confirms the hypothesis that due to the two separate size scales of active nucleation site on nanocavity surfaces and porous pillar surfaces, the HTC could be further enhanced without compromising to each other on a combined surface. Hence, the HTC on a combined surface can be represented by Equation 9, which is a simple superposition of Equations 2 and 8 representing the two active nucleation site ranges from nanocavities and porous pillars:


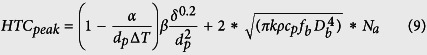


## Conclusion

Based on the comparisons among experimental data of this study and previous reportes by other research groups, and predictions of theoretical models developed in this study and previously reported, it demonstrates that different physics of the liquid/solid/vapor interaction at different spatial scales exist on spherical Cu nanocavity surfaces and porous Cu pillar surfaces. For CHF enhancement, spherical Cu nanocavity surfaces control the hydrodynamic instability wavelength through reduced contact angles, while porous Cu pillar surfaces control hydrodynamic instability wavelength by replacing Kevin-Helmholtz wavelength with module wavelength. Furthermore, the CHF will be dominated by the modulated wavelength of porous pillars over contact angle on an integrated surface of spherical Cu nanocavity coated porous Cu pillars when the characteristic length of surface structure is higher than the critical length of ¼ Kevin-Helmholtz wavelength or ¼ module wavelength. Both spherical Cu nanocavity surfaces and porous Cu pillar surfaces have greatly increased pool boiling HTC by individually increasing the active nucleation site densities, and an integrated surface has an even higher HTC enhancement, which is a sum of HTC enhancements on both nanocavity surface and porous pillar surface, by all active nucleation site densities on both surfaces. This collective role from both nanostructures and porous pillars is due to the fact that two structures offer active nucleation sites in two totally separate length scales, one is in nanometer scale and the other is in micron scale. The peak HTC on an integrated surface can be approximated as a simple superposition of HTCs on a nanostructure surface and a porous pillar surface.

As a conclusion, it has been proven that macroscale pattern, microscale pore, and nanoscale cavity can be effectively integrated to improve pool boiling heat transfer. The CHF on an integrated multiscale structure is decided by the macroscale structures, while the HTC can take full advantage of enhancements from both microscale and macroscale structures.

## Methods

### Synthesis of Spherical Cu nanocavity Surfaces

Initially, copper substrate surfaces are wet sanded using 600 grit sand paper, and then a final polishing was conducted in two steps using 1.0 and 0.5 μm Alpha Alumina particles (Sigma-Aldrich, Inc., USA) mixed at a 1:10 ratio with distilled water with a Spectrum System 1000 polisher (LECO, USA). Polystyrene spheres of 200 nm and 500 nm diameter (Sigma-Aldrich, Inc., USA) were electrophoretically deposited into colloidal crystals onto the polished copper substrates to form templates under a constant potential difference of 25 V for 1 hour. Spherical Cu nanocavities were synthesized by a potentiostatic deposition method (VersaSTAT IV, PAR Inc., USA) in a bath of 0.5 M copper sulfate solution (Sigma-Aldrich, Inc., USA) at a constant potential of −0.5 V.

### Synthesis of Porous Structures

The sintering process began with 0.65 g of 600 μm copper particles obtained from ACU Powder International filling a graphite mold. A copper test bar was then polished and inserted into the mold. A pressure of 150 MPa was applied using the four stainless steel bolts on supporting stainless steel plates. The tube furnace was used and initially purged with grade 4 hydrogen and argon for 15 minutes to remove all air from the system. The argon flow remained constant throughout the entirety of the experiment, while the hydrogen flow was shut off once the furnace reached a temperature of 400 °C. The temperature controller program gradually raised the furnace temperature to 700 °C over 14 hours, dwelled at that temperature for two hours, and then ramped back to room temperature over 14 hours.

### Synthesis of Integrated Porous Structures with Nanocavities

After the porous structures were sintered, the same procedure was applied as described in the synthesis of nanostructures section. The integrated structures were examined by SEM to characterize both nanostructures and porous structures. Altogether, thirteen individual surfaces were tested and characterized.

### Contact angle measurement

The contact angle goniometer used in this study was a Surface Electro Optics Contact Angle Analyzer paired with Surfaceware 7 software for data analysis. The system consisted of a Thorlabs CMOS Camera featuring a Navitar High Zoom Lens and high speed image capture card. To measure the equilibrium contact angle, a droplet was dropped onto the surface by a step-motor pump. Once the droplet had settled, a picture was taken using the high zoom camera, and the picture was imported into Surfaceware 7. The program then identified the edges of the droplet and measured the contact angle. To measure the advancing contact angle, a water droplet size was increased by actuating a step-motor pump, causing the droplet to grow and spread across the surface. A video sequence was taken, and freeze frames at different points in the video were used to identify the advancing contact angle when the triple-phase contact line was about to move. Receding contact angle was measured in a similar manner. The step-motor pump was used to remove the liquid from the droplet, and the droplet began to shrink. an image at this moment was taken at the moment the triple phase contact line was about to move. And this image was used by Surfaceware 7 to determine the receding contact angle as previously stated. Measurements were conducted using a 2.5-μ L droplet of DI water, and were repeated ten times for averaging characteristics in each measurement.

### Pool boiling experiments

For boiling performance evaluation, we conducted pool boiling experiments using the in-house made apparatus setups as reported previously. Deionized (DI) water was used as the working fluid, and all experiments were conducted at saturation conditions under ambient pressure (i.e., 100 °C of DI water at 1 atm). A detailed description of the experimental setup and procedure can be found in literature^26^,^35^.

## Additional Information

**How to cite this article**: Li, C. H. and Rioux, R. P. Independent and collective roles of surface structures at different length scales on pool boiling heat transfer. *Sci. Rep*. **6**, 37044; doi: 10.1038/srep37044 (2016).

**Publisher's note:** Springer Nature remains neutral with regard to jurisdictional claims in published maps and institutional affiliations.

## Figures and Tables

**Table 1:** Equilibrium, advanced, and receding contact angles.

Name	Pore Size, (nm)	Thickness (μm)	Equilibrium θ_e_	Advancing θ_a_	Receding θ_r_
Polished	N/A	N/A	78.35°	85.65°	40.01°
200	200	5	112.17°	130.23°	36.4°
500	500	5	122.41°	146.44°	35.48°

**Table 2:** Comparison among experimental and theoretic CHFs.

Surface	Experimental CHF (W/cm^2^)	Liaw and Dhir, (W/cm^2^)	Kandlikar Prediction. (W/cm^2^) (Kandlikar, 2001)	Chu Prediction. (W/cm^2^) (Chu *et al*. 2012)
Polished	116.34	57/69	133.11	89.75
